# A Letter to the Reverend Thomas Rennell, Concerning His Remarks on Scepticism, from a Graduate in Medicine, of the University of Oxford

**Published:** 1819-07-01

**Authors:** 


*A Letter to the Reverend* Thomas Rennell, *concerning his Remarks on Scepticism, from a Graduate in Medicine, of the University of Oxford.* Octavo, sewed, pp. 60. London, 1819.

This little Tract is from the pen of a young physician in the west end of the town, who is already known to the public by some ingenious observations on the pathology of irregular manifestations of the mind. "It professes to point out errors, and only errors, in a work, which contains much that is valuable and interesting. But this is not all: its author professes the sincerest friendlyness to those principles which Mr. Rennell vindicates."

It was natural to expect that Mr. Rennell would commit some oversights in pathology, and even in physiology. "Now, Sir, it is my intention in the following pages, as far as in my power, and as having a common object with yourself, to anticipate the adversaries of your religious principles in pointing out the errors of your physiological reasoning, and to suggest that, these errors granted, the principles themselves remain unshaken." P. 3.

Notwithstanding this, and several other professions of friendship in the work under review, we are not quite satisfied, in our own minds, that the friendship is *sincere*. We have been led to entertain *misgivings* of this kind, from the tone and manner in which the Graduate has turned some of the positions of the Divine. Yet we may be wrong; and if so, we beg the Physician's pardon. We agree with him in thinking, that Mr. Rennell has not sufficiently admitted the intimate connexion and mutual dependence of mind and matter, in this state of our existence, which is so well expressed by the Heathen Philosopher—

*Mens* agitat molem, et magno se corpore *miscet:*

P. S. We have just learned [May 5th.] that Mr. Lawrence has suppressed his "Lectures on the Natural History of Man." This, we conceive, is a wise sacrifice, on his part, to the general sense, as well as the general good of society. We are not among those who insult the feelings of the afflicted—our maxim being—

Parcere subjectis, et debellare superbos.

Our remarks on Mr. Rennell's work, in the first Article of this Number, were written before Mr. Lawrence's work was published. They are directed against principles and not against persons. They record our abhorrence of the former, without diminishing our respect for the professional talents, or private character of the *latter*.

